# Buccal versus skin graft for two-stage repair of complex hypospadias: an Egyptian center experience

**DOI:** 10.1186/s12894-022-01069-6

**Published:** 2022-07-26

**Authors:** Salah E. Shebl, Mabrouk M. Akl, Mohamed Abdalrazek

**Affiliations:** 1 Urology Department, Al Zahraa University Hospital, Faculty of Medicine for Girls, Al-Azhar University, Abbasia, Cairo, Egypt; 2 Pediatric Surgery Department, Faculty of Medicine Al-Azhar University, Cairo, Egypt

**Keywords:** Complex hypospadias, Two-stage repair, Buccal graft, Skin graft

## Abstract

**Background:**

Urethral reconstruction in complex hypospadias poses a significant challenge. We report our 10-year experience with buccal mucosa graft (BMG) in the two-stage repair of complex hypospadias and compare its results to the skin graft.

**Methods:**

We retrieved the data of 15 patients with complex hypospadias who underwent two-stage repair using the BMG at our institution. The data were compared to 13 patients who underwent skin graft during the same period.

**Results:**

The median follow-up duration was 14 (12–17) months in the BMG group and 16 (13.5–22.5) months in the skin graft group. Patients in the BMG had a numerically lower incidence of the diverticulum, wound dehiscence, fistula, and infection than the skin graft group, however, without statistically significant difference (*p* > 0.05). On the other hand, the incidence of meatal stenosis and urethral stricture was significantly lower in the BMG group (0% each) compared to the skin graft group (30.8% each; *p* = 0.02). At the same time, there were no reported cases of graft contracture. The frequency of donor site morbidity was significantly higher in the skin graft group compared to the BMG group (*p* = 0.003). The BMG led to a lower incidence of postoperative straining than the skin graft (0% vs. 38.5%, *p* = 0.03). Only one patient needed revision surgery after skin graft, compared to no case in the BMG (*p* = 0.27).

**Conclusion:**

The present study demonstrates the feasibility and durable outcomes of the BMG in the setting of two-stage repair of complex hypospadias.

## Introduction

Hypospadias affects up to 0.4% of the global male population, which is expected to rise in the upcoming years. Previous epidemiological figures highlighted a notable geographic variation in the incidence of hypospadias, being highest in North America and lowest in Asia [1, 2]. The disorder encompasses several abnormalities that include ectopic ventral urethral opening, alongside variable degrees of chordee, ventral penile curvature, and abnormal urethral plate width [3]. Hypospadias can exert a considerable burden on the healthcare system and negatively affect the quality of life of the patients; besides, patients with hypospadias experience difficulties in urination and fertility issues [4]. Thus, the primary goal of treatment is to restore the normal cosmetic and functional features. Several surgical approaches are proposed for hypospadias repair.

Two-stage hypospadias repair is commonly indicated for patients needing revision surgery due to one-stage repair’s shortcomings in terms of scarred/replaced urethral plate and slower healing rate [5]. The two-stage repair typically involves harvesting a free graft from the inner prepuce [6]. However, a subset of the patients may still suffer from functional limitations and grade III complications after several surgeries, named “complex hypospadias.” In such cases, shortage or residual contracture of penile skin can be present, making it unsuitable for harvesting [7]. Both skin and buccal mucosal grafts (BMG) represent suitable alternatives for inner prepuce graft. While BMG was associated with satisfactory functional and cosmetic outcomes, the technique requires advanced surgical skills that may not be available in many local settings [5]. On the other hand, skin graft from the groin is feasible in many local settings; however, it poses the shortcomings of coarse hair growth and donor site morbidity. This report demonstrated our 10-year experience with BMG in the two-stage repair of complex hypospadias and compared its results to the skin graft.

## Methods

The protocol of the current trial was approved by the local ethics committee of Quality Education Assurance Unit, Al-Azhar Faculty of Medicine, Nasr city, Cairo, Egypt (Registration Number: ped_1. 16Med._00016). All procedures run in compliance with the standards of the Declaration of Helsinki [8]. Due to the retrospective nature of the study, the need for written informed consent was waived.

### Study design and patients

In this retrospective chart review, the total number of hypospadias repairs in the study period were 1652 patients. BMG grafts in total were 82. We retrieved the data of all 15 patients with complex hypospadias who underwent two-stage repair using the BMG at Al-Azhar University Hospitals through the period from January 2010 to December 2020. In addition, we retrieved the data of all 13 patients who underwent skin graft during the same period. Patients with incomplete records of postoperative data or follow-up duration of less than 6 months were excluded. Patients from both groups were chosen consecutively from all available data records.

### Data collection and procedures

The following data were retrieved from the patients’ records: age, previous surgeries, presence of preoperative penile torsion, type of hypospadias at previous surgery, previous graft, graft characteristics (length, width, and donor site complications), site of the meatus, type of the second layer, pre and postoperative dripping and straining, pre and postoperative penile curvature or chordae, pre and postoperative meatal stenosis, postoperative spraying of micturition, diverticulum, postoperative wound dehiscence, fistula infection, urethral stricture, graft contraction, need for revision surgery, type of the second layer.

All patients underwent general anesthesia and procedures were performed in supine position. The choice of the surgical technique was per the surgeon’s preference. A total of 15 patients underwent a two-stage repair with BMG graft in the first stage followed by second stage urethroplasty later with local tissue flap (tunica vaginalis or subcutaneous dartos flap) as a second layer coverage of the new urethra. Tunica vaginalis flap was used in five cases, and subcutaneous dartos flap was used in the remaining ten cases. The BMG was harvested in all cases from the inner check by the technique described by Eppley et al. [9]. Briefly, a local anesthetic was infiltrated on the oral mucosa, followed by a sharp dissection of the mucosal graft, leaving the muscle intact; the incision was closed by continuous suture. On the other hand, the skin graft was harvested from the inguinal region at McBurney’s point in 13 cases, with tunica vaginalis flap used in six cases and subcutaneous dartos flap used in seven cases. Only Vaseline dressing is used and fixed over the postoperative graft with the Foley catheter passing out of the dressing for 5–7 days. This is removed along with the Foley catheter. The frequent moist dressing is kept until complete healing and appropriate for second stage. The success was defined as a straight penis with meatus at the tip of the glans, without functional complications that need reoperation procedures.

### Study’s outcomes

The primary outcome of the present study was to compare the rate of late and postoperative complications between BMG and skin graft. The secondary outcome was to compare the two grafts in terms of success and donor site complications.

### Statistical analysis

Retrieved data were summarized and processed with IBM SPSS statistical software (version 25). Frequencies and summary statistics were used to describe continuous and categorical data, respectively. The hypothesize of significant difference between the two types of graft in terms of postoperative complications was tested using the Chi-square test, with Fisher exact whenever needed. The association between the type of graft and continuous data was tested using the Mann–Whitney test. *p*-value < 0.05 was regarded as statistically significant.

## Results

The mean age of the included patients was comparable between BMG and skin graft groups (*p* = 0.52). The majority of the patients in both groups had two previous surgeries (40% vs. 38.5%, respectively, *p* = 0.97). The hypospadias was mostly mid-shaft or penoscrotal in both groups (*p* = 0.81). Patients in the skin graft group had numerically higher rates of penile torsion (30.8%) and meatal stenosis (30.8%); however, without significant difference (*p* > 0.05). On the other hand, 60% of the BMG group had penile curvature or chordae, compared to 30.8% in the skin graft group (*p* = 0.12). The frequency of preoperative urination difficulties was comparable between both groups. Likewise, the graft length and width were similar between the BMG and skin graft groups (Table 1).

**Table 1:** Preoperative and graft characteristics of the included patients

Variables	BMG (n = 15)	Skin graft (n = 13)	*p*-value
Age in years, median (IQR)	4.5 (4–10)	6 (3.8–11.5)	0.52
Number of prior surgeries, No. (%)			0.97
One	5 (33.3)	4 (30.8)	
Two	6 (40)	5 (38.5)	
Three	4 (26.7)	4 (30.8)	
Type of hypospadias, No. (%)			0.81
Distal	5 (33.3)	5 (38.5)	
Mid-shaft	2 (13.3)	1 (7.7)	
Penoscrotal	4 (26.7)	5 (38.5)	
Proximal	4 (26.7)	2 (15.4)	
Penile torsion, No (%)	2 (13.3)	4 (30.8)	0.26
Penile curvature or chordae, No (%)	9 (60)	4 (30.8)	0.12
Meatal stenosis, No (%)	1 (6.7)	4 (30.8)	0.09
Previous skin graft, No (%)	3 (20)	0	0.09
Urination difficulties, No (%)			
Dripping	2 (13.3)	3 (23.1)	0.51
Straining	1 (6.7)	3 (23.1)	0.11
Graft length in cm, median (IQR)	5 (4–5)	5 (4 -6)	0.86
Graft width in cm, median (IQR)	1.5 (1–2)	1.5 (1.3–2)	0.99

The median follow-up duration was 14 (12–17) months in the BMG group and 16 (13.5–22.5) months in the skin graft group (*p* = 0.34). Concerning early complications, patients in the BMG had a numerically lower incidence of the diverticulum (0% vs. 7.7%), wound dehiscence (0% vs. 15.4%), fistula (13.3% vs. 23.1%), and infection (13.3% vs. 30.8%) than the skin graft group, however, without statistically significant difference (*p* > 0.05). On the other hand, the incidence of meatal stenosis and urethral stricture was significantly lower in the BMG group (0% each) compared to the skin graft group (30.8% each; *p* = 0.02). At the same time, there were no reported cases of graft contracture. The frequency of donor site morbidity was significantly higher in the skin graft group compared to the BMG group (P = 0.003). The BMG led to a lower incidence of postoperative straining than the skin graft (0% vs. 38.5%, *p* = 0.03). Only one patient needed revision surgery after skin graft, compared to no case in the BMG (*p* = 0.27), Table 2.

**Table 2:** Postoperative outcomes of the studied groups

Variables	BMG (n = 15)	Skin graft (n = 13)	*p*-value
Follow-up in months, median (IQR)	14 (12–17)	16 (13.5–22.5)	0.34
*Early complications, No (%)*			
Diverticulum	0	1 (7.7)	0.27
Wound dehiscence	0	2 (15.4)	0.12
Fistula	2 (13.3)	3 (23.1)	0.51
Infection	2 (13.3)	4 (30.8)	0.26
*Late complications, No (%)*			
Meatal stenosis	0	4 (30.8)	0.02
Residual chordae	2 (13.3)	4 (30.8)	0.27
Urethral stricture	0	4 (30.8)	0.02
Graft contracture	0	0	–
Donor site complications			0.003
Keloid	0	1 (7.7)	
Lip numbness	1 (6.7)	0	
Scar	1 (6.7)	8 (61.5)	
*Urination difficulties, No (%)*			
Dripping	0	3 (23.1)	0.17
Straining	0	5 (38.5)	0.031
Spraying	1 (6.7)	4 (30.8)	0.09
Need for revision surgery, No (%)	0	1 (7.7)	0.27

## Discussion

Urethral reconstruction in complex hypospadias poses a significant challenge. In the setting of two-staged repair, a free graft from the inner prepuce is the preferred type of graft for complex hypospadias. Nonetheless, a subset of the patients become unsuitable for inner prepuce graft due to repeated surgeries and residual contracture of penile skin. In such cases, the BMG and skin graft can be employed for hypospadias repair. Skin grafts were historically employed in two-stage urethroplasty due to their abundancy and being easy to harvest. However, skin graft is liable to contracture and thickness when harvested due to the keratinized epithelium and the split-thickness, leading to stricture [10]. Besides, the presence of skin hair limits its use for urethral reconstruction [9]. Thus, BMG has replaced skin graft as the gold standard graft for two-staged repair in many centers. The BMG represents a reliable option with well-established long-term durability and lower incidence of postoperative complications, compared to skin graft. In addition, BMG minimizes the time to harvest and reduces the risk of recurrence [11]. However, only few reports compared the postoperative outcomes between the BMG and skin graft. This report demonstrated our 10-year experience with BMG in the two-stage repair of complex hypospadias and compared its results to a historical cohort with skin graft. The skin graft was performed in a historical cohort before the wide use of BMG and its use in complex hypospadias our center.

Patients undergoing hypospadias repair are prone to wide range of early and postoperative complications, whose risks are significantly increased in patients undergoing multiple surgeries. Previous reports demonstrated that patients with hypospadias repair can develop acute postoperative complications in the form of infection, wound dehiscence, fistula, penile malformation, hemorrhage, and flap necrosis [12]. Such complications were reported to occur more frequently in patients undergoing staged surgery [13]. Previous reports showed that BMG urethroplasty has an acceptable postoperative safety profile, with few rates of acute postoperative complications; nonetheless, patients with complex stricture of penile urethra—a frequent finding in patients with complex hypospadias—had higher risk of postoperative complications following BMG urethroplasty [14]. On the other hand, skin graft urethroplasty is usually associated with higher risk of acute postoperative infection and fistula formation [15]. In the present report, we found that patients in the BMG had a numerically lower incidence of the diverticulum, wound dehiscence, fistula, and infection than the skin graft group, however, without statistically significant difference (*p* > 0.05). To our knowledge, this is the first study that compared the incidence of acute postoperative outcomes between BMG and skin graft two-staged hypospadias repair. However, our findings concerning the rate of acute postoperative outcomes following BMG run in line with previous reports demonstrating low incidence of infection, wound dehiscence, fistula, and bleeding among patients with repeated surgery [16–19].

## Late complications

According to Bracka, the use of extra-genital skin graft in the setting of two-stage hypospadias repair should be discouraged due to the possibility of hair growth and high incidence of donor site morbidity, including keloid scars [5]. On the other hand, BMG is the most preferred oral mucosa for two-staged hypospadias repair as it is typically devoted of scaring or significant donor site morbidity. While labial mucosa may lead to lip distortion and oral contracture [20]. In the preset study, we found that the frequency of donor site morbidity was significantly higher in the skin graft group compared to the BMG group (*p* = 0.003).

The present study poses certain limitations. The study was retrospective in nature, which increases the risk of ascertainment and selection bias. Besides, the study was limited to a single center only with a small sample size, which may affect the generalizability of our findings. The follow-up duration was also limited to a maximum of 2 years, and there were no available data to assess the long-term outcomes of the BMG and skin graft beyond the 2 years of follow-up. Lastly, the BMG was performed by more than one surgeon; it was previously reported that the outcome of the BMG depends on the surgeon’s skills and years of experience with the technique [5]. The study also used subjective outcome measures.

In conclusion, the present study demonstrates the feasibility and durable outcomes of the BMG in the setting of two-stage repair of complex hypospadias. Our results showed the onlay BMG urethroplasty was associated with lower incidence of postoperative complications and donor site morbidity than the skin graft urethroplasty. Besides, the onlay BMG urethroplasty significantly improved the symptoms of urination difficulty. Thus, onlay BMG urethroplasty should be preferred over the skin graft in centers with experience in onlay BMG urethroplasty. Further studies are warranted to confirm our findings and identify the subgroup of patients who are suitable candidate for onlay BMG urethroplasty.

## Data Availability

The dataset generated and or analyzed during the current study are not publicly available to maintain patient and institution privacy but are available from corresponding author on reasonable request.

## Abbreviation

BMG: Buccal mucosal grafts
